# CNS Aspergillosis: A Downside of Corticosteroid Use

**DOI:** 10.7759/cureus.62018

**Published:** 2024-06-09

**Authors:** Pranay R Bonagiri, Anjalee Raman, Sharjeel Hassan, Andrea Ramsey

**Affiliations:** 1 Internal Medicine, Scripps Mercy Hospital, San Diego, USA; 2 Internal Medicine, Touro University California, Vallejo, USA; 3 Infectious Disease, Scripps Mercy Hospital, San Diego, USA

**Keywords:** seizure, steroids, glucocorticoids, aspergillus abscess, aspergillus

## Abstract

Glucocorticoids are ubiquitously used by physicians for a myriad of diseases. Though powerful and potentially lifesaving, sometimes the dangerous side effects are not at the forefront of our medical decision-making. By immunosuppressing patients, glucocorticoids can place patients at increased risk for not only the metabolic effects of chronic glucocorticoid use but also increased risk for opportunistic infections. Patients at increased risk include those on prolonged courses or those that require high doses. We report a case of a 34-year-old man who was initiated on glucocorticoids for an unknown rheumatologic disease and presented with generalized weakness, fatigue, nausea, and vomiting. The patient experienced a seizure, which prompted head imaging. A mass was found and eventually biopsied, which was notable for *Aspergillus fumigatus.* The patient was initiated on antifungals for CNS aspergillosis and recovered.

## Introduction

Aspergillus is a large genus of fungus that is important to be aware of due to its ubiquity in nature [1-2]. *Aspergillus fumigatus*, specifically, plays a central role in the pathogenesis of aspergillosis [3]. Infections most commonly occur in immunocompromised individuals, though there is growing awareness of infection in immunocompetent individuals. One of the most common causes of immunosuppression is glucocorticoids. Though a powerful medication, the side effects of glucocorticoids are often in the background of our medical decision-making. In this case report, we present a 34-year-old patient with an unknown rheumatologic disease experiencing one of the dangerous side effects of glucocorticoid use.

## Case presentation

**Figure 1:**
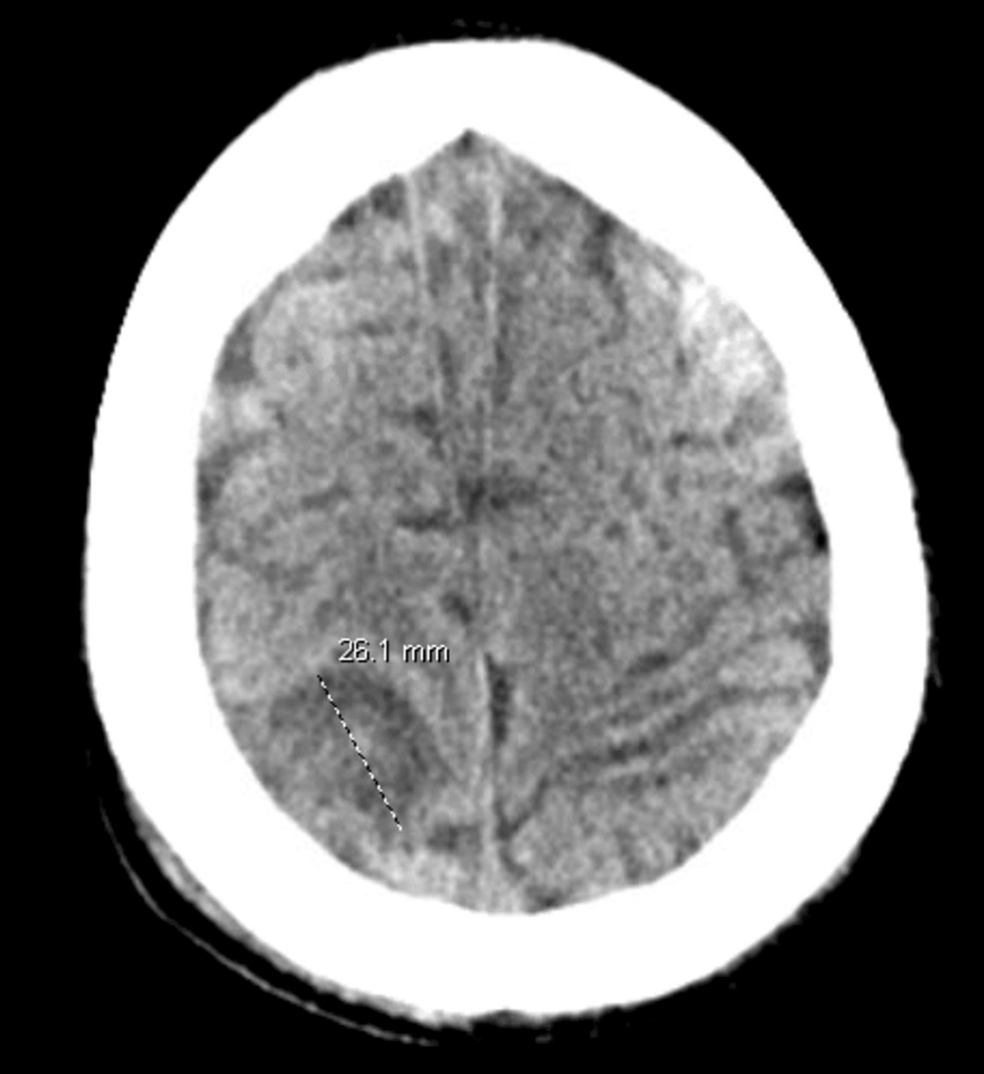
Axial view of non-contrast CT head with 2.6cm rounded area of hypoattenuation with mild mass effect in the posterior right frontal lobe

**Figure 2:**
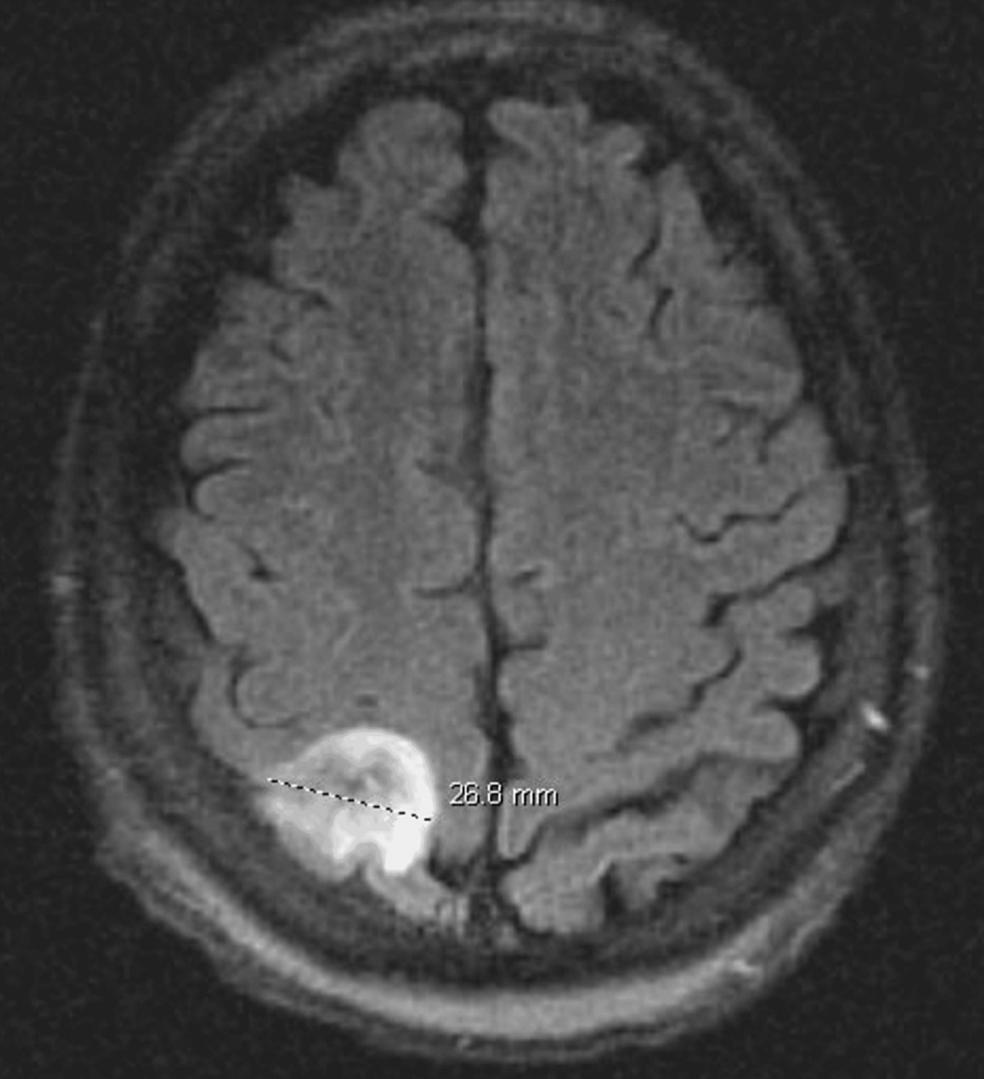
T2/FLAIR hyperintense lesion in R postcentral gyrus with minimal central restricted diffusion and mild associated surrounding vasogenic edema FLAIR: fluid-attenuated inversion recovery

A 34-year-old man with an unclear rheumatological history presents with weakness, fatigue, nausea, and vomiting for one week which has progressively worsened. On presentation, the patient was largely unresponsive, and the history was obtained from the family. His past medical history was notable for chronic prednisone use of unclear duration. He was prescribed 7.5mg daily; however, he started to take doses of at least 20mg as he felt the low dose was not working. In the emergency department, he developed sepsis with rigors, tachypnea with respiratory rates in the 40s, tachycardia with heart rates in the 120s, fever of 101.3, and hypotension with blood pressure in the 80s/50s. Imaging was notable for multifocal pneumonia with *Klebsiella pneumoniae *bacteremia. The patient also developed acute tubular necrosis and atrial fibrillation with rapid ventricular response most likely secondary to sepsis. During his hospitalization though, he experienced a seizure, the exact type was not specified. EEG was negative for epileptiform discharges, but notable for mild to moderate generalized slowing. The patient was started on levetiracetam and did not experience any other seizures through his hospital course. CT head was notable for a new right 2.6cm posterior superior parietal lobe mass (Figure 1) and MRI brain demonstrated the 2.6cm T2/fluid-attenuated inversion recovery (FLAIR) hyperintense lesion in R postcentral gyrus (Figure 2).

**Figure 3:**
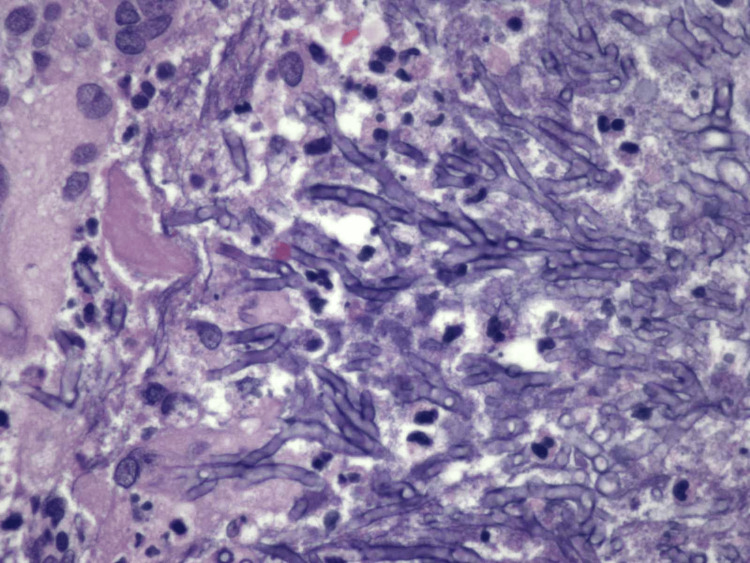
Septate hyphae with acute angle branching within the granuloma

Initially, this lesion was attributed to septic emboli in the setting of *Klebsiella bacteremia*; however, repeat imaging while in-hospital approximately six weeks later showed no improvement on systemic therapies. Neurosurgery performed an excisional biopsy of the brain lesion, which was notable for suppurative granulomas with fungal organisms with septate hyphae and acute-angle branching, ultimately determined to be *A. fumigatus *(Figure 3).

The strain was resistant to fluconazole. After an 80-day hospital stay, the patient was discharged on anti-epileptic medication as well as voriconazole for nine months. Repeat brain imaging is pending.

## Discussion

Aspergillus is a large genus with over 800 separate species [1]. Aspergillosis is usually caused by *A. fumigatus*, but other responsible organisms include *A. flavus*, *A. terreus*, *A. niger*, *A. nidulans*, *A. ustus*, and *A. oryzae*. *A. fumigati* is an important organism to be aware of due to its ubiquity. A saprophytic fungus found commonly in soil, *A. fumigatus* is easily aerosolizable and can be found in concentrations up to 100 conidia per m^3 commonly [2]. *A. fumigatus*, among other fungi, is present in the physiological lung microbiome in healthy patients and those with chronic lung disease [3].

Aspergillus can cause a wide spectrum of diseases from hypersensitivity/allergy diseases, structural lung diseases with/without inflammation, and finally invasive aspergillosis in the lung or extra-pulmonary [2]. Extra-pulmonary manifestations are rare but have been reported in many different organ systems. These include endocarditis, sino-nasal involvement, cerebral involvement, splenic infarcts/abscesses, renal infection, gastrointestinal aspergillosis, osteomyelitis, arthritis, subacute thyroiditis, and even endophthalmitis [4]. For CNS aspergillosis, spread usually occurs either through hematogenous spread or direct invasion through the sinuses or nasopharyngeal tract [4,5]. Interestingly, direct spread seems to be more common in immunocompetent patients as opposed to hematogenous spread [5,6]. Perhaps, immunocompetent hosts are able to better protect against vascular invasion, so the fungi only can spread locally.

CNS aspergillosis should be kept in the differential for any immunocompromised patients as neurologic symptoms can be nonspecific including headache, altered mental status, seizures, and focal neurologic deficits [5]. This differential can be quite large, but specifically in immunocompromised patients the following should be considered: bacterial abscess, CNS lymphoma, toxoplasmic encephalitis, and tuberculoma. Corticosteroids appear to be the most common risk factor [6]. The fungi have a predilection for invasion of vessel walls, both large and small vessels, resulting in thrombosis complicated by infarction and hemorrhage [5]. As the brain tissue is weakened, Aspergillus can further invade past vessel walls and form abscesses [5]. Even though vascular invasion seems to be common, true mycotic aneurysms (infection of the arterial wall itself) seem to be rare as they seem to have a preference for the brain rather than vascular tissue [5].

Imaging, either MR or CT, can be notable for multiple lesions that present as abscesses, infarctions, or hemorrhages [5]. All parts of the brain can be affected; however, there appears to be a predilection for the corticomedullary junction in the supratentorial area [7]. Additionally, the lesions tend to contrast enhancing, more likely annular than nodular, peripheral enhancement on T1, and hypointense rims on T2 [7]. Our patient presented with an infarction that was ultimately determined to be a heterogeneous mass of fungal etiology.

Given his immunosuppressed status, CNS aspergillosis should have likely been considered earlier during his hospitalization. Etiologies of brain mass for immunocompetent patients should be considered as well including primary or metastatic brain tumors, vascular anomalies, and infections (such as bacterial abscesses). Timely recognition can prevent morbidity. Antifungal treatment can vary but include azoles, echinocandins, and amphotericin B. Thankfully, the strain of *A. fumigatus* in our patient was susceptible to some azoles, but the proportion of azole-resistant *A. fumigatus* is rising throughout the world [4].

## Conclusions

CNS aspergillosis is an important complication to be aware of when caring for patients with chronic glucocorticoid use. Clinical presentation can vary, but unexplained headaches, altered mental status, and/or seizures should be investigated with head imaging. Biopsy and histologic diagnosis play a central role in diagnosis and treatment, as there is a rising incidence of azole-resistant Aspergillus.
